# Changes in Group A *Streptococcus* *emm* Types Associated with Invasive Infections in Adults, Spain, 2023

**DOI:** 10.3201/eid2911.230857

**Published:** 2023-11

**Authors:** Alba Bellés-Bellés, Núria Prim, Saray Mormeneo-Bayo, Pilar Villalón-Panzano, Mónica Valiente-Novillo, Alfredo Jover-Sáenz, Núria Aixalà, Albert Bernet, Éric López-González, Ivan Prats, Mercè García-González

**Affiliations:** Hospital Universitari Arnau de Vilanova, Lleida, Spain (A. Bellés-Bellés, N. Prim, S. Mormeneo-Bayo, A. Jover-Sáenz, N. Aixalà, A. Bernet, É. López-González, I. Prats, M. García-González);; Institut de Recerca Biomèdica de Lleida, Lleida (A. Bellés-Bellés, N. Prim, S. Mormeneo-Bayo, A. Jover-Sáenz, A. Bernet, É. López-González, I. Prats, M. García-González);; Centro Nacional de Microbiología, Instituto de Salud Carlos III, Madrid, Spain (P. Villalón-Panzano, M. Valiente-Novill)

**Keywords:** *Streptococcus pyogenes*, bacteria, streptococci, *emm* typing, invasive infection, group A Streptococcus, Spain

## Abstract

An increase in invasive group A *Streptococcus* infection was detected in the northeast of Spain in November 2022. A postpandemic decline in the diversity of circulating *emm* types involved in invasive group A *Streptococcus* was observed, along with the emergence of *emm*49 in this geographic area.

*Streptococcus pyogenes* (group A *Streptococcus *[GAS]) can cause a broad range of infections. Although usually associated with streptococcal pharyngitis and skin and soft tissue infections, GAS can also cause life-threatening infections, such as sepsis or necrotizing fasciitis (*1*).

In December 2022, the World Health Organization issued an alert about increasing rates of invasive GAS (iGAS) infections in children in Europe (*2*). This warning was followed by a health advisory from the US Centers for Disease Control and Prevention that reported an increase in these infections among children in the United States (*3*). Since then, several countries in Europe have notified an increase, particularly in the pediatric population (*4*).

A total of 31 culture-confirmed iGAS cases (incidence 0.0912 cases/1,000 inhabitants) were detected during November 2022–May 2023 in the province of Lleida (catchment of 340,000 inhabitants) in northeast Spain. Invasive cases were defined according to Centers for Disease Control and Prevention definitions (*5*). The median age was 62 years (range 3–93 years); 4 cases occurred in children. Three deaths in adults were notified during this period. Given that increase, we analyzed the distribution of iGAS in Lleida province during January 2011–May 2023 (Appendix Figure). The average number of iGAS cases per year was 5.2 (incidence 0.0153 cases/1,000 inhabitants/year) during 2011–2018. A total of 19 culture-confirmed cases (incidence of 0.0559 cases/1,000 inhabitants/year) were reported in 2019, followed by a decline in incidence during the COVID-19 pandemic (0.0162 cases/1,000 inhabitants/year). The increase detected since November 2022 surpassed 2019 incidence.

Hospital Universitari Arnau de Vilanova (Lleida, Spain) began participating in the iGAS national surveillance program in April 2019. Conducted at Centro Nacional de Microbiología (Majadahonda, Spain), this program performs *emm* typing and toxin gene profiling of iGAS isolates collected from microbiology laboratories. We analyzed the monthly distribution of *emm* types involved in 61 iGAS cases reported during January 2019–May 2023 in our geographic area (Figure). Overall, 19 different *emm* types were detected; *emm*1 (n = 18/61) was the most frequent, followed by *emm*49 (n = 8/61) and *emm*89 (n = 8/61). A decrease in type diversity was observed since November 2022; the 6 *emm* types detected were *emm*1 (n = 15/31), *emm*12 (n = 5/31), *emm*49 (n = 5/31), *emm*89 (n = 4/31), *emm77* (n = 1/31) and *emm*58 (n = 1/31) (Table). 

**Figure:**
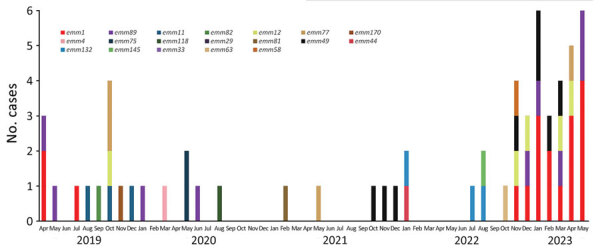
*emm* type distribution of 61 culture-confirmed invasive group A *Streptococcus* infections, by month, Lleida, northeast Spain, January 2019–May 2023

A large increase in iGAS was detected beginning in November 2022. Decreased exposure to GAS during the COVID-19 pandemic because of mask use and social isolation might have contributed to its low circulation. The decline of masking and social distancing, combined with high rates of respiratory viruses during the winter of 2021–2022, might be behind the increase in iGAS. We found that most iGAS occurred in adults and had a low mortality rate, in contrast with previous reports (*4*).

Certain GAS *emm* types have been strongly associated with invasive infections. However, a notorious geographic diversity in the circulation of *emm* types exists (*7*). The most common types involved in iGAS in Spain from 2007–2019 were *emm*1, *emm*89, and *emm*3, according to the GAS Surveillance Program in Spain. In contrast, only 1 *emm*49 isolate was detected during that period (*6*). In our study, *emm*1 was predominant in accordance with other reports in Europe (*4*). After *emm*1, *emm*49 and *emm*89 were the most frequent *emm* types in our study. A substantial expansion of *emm*49 has been recently reported in United States, where it represents the third most common *emm* type associated with iGAS, after *emm*1 and *emm*89 (*8*). However, *emm*49 was an unusual *emm* type in northeast Spain until recently and was rarely involved in iGAS in Europe (*4*,*9*). After *emm*49 was first detected in the nationwide surveillance program in Spain in 2011, circulation increased in Spain during 2021–2023 (P. Villalón-Panzano, unpub. data). Including our 8 cases, 11 *emm*49 iGAS cases were notified by this program during this period. Despite the lack of a clear epidemiologic link, an outbreak could not be ruled out in this area. The first detection of *emm*49 iGAS in Lleida province was in October 2021. The main clinical manifestations of iGAS associated with *emm*49 were skin and soft tissue infections, as previously described (*8*).

A high incidence of iGAS in children during 2019 was recently reported in Spain (*10*). Our data showed an increasing trend of iGAS in adults in our area that was interrupted by the COVID-19 pandemic. Together with an accumulation of iGAS, a decline in *emm* type diversity has been detected since the World Health Organization alert in late 2022.

In conclusion, this report supports that the increase in iGAS in adults probably began before the COVID-19 pandemic in northeast Spain. The postpandemic increase was caused by a limited number of *emm* types. Although *emm*1 was the most common type, *emm*49 is an emergent cause of iGAS in Europe. Continuous surveillance is essential to detect the emergence and spread of *emm* types associated with iGAS in different geographic areas.

**Table:** Phenotypic and genotypic characteristics of 61 *Streptococcus pyogenes* isolates in invasive infections and clinical manifestations, Lleida province, northeast Spain, January 2019–May 2023*

***emm* type, n = 61**	*emm* cluster†	Exotoxin gene profile	Resistance phenotype	Clinical manifestations
***emm*1, n = 18**	A-C3	A, G, J, *smeZ*, n = 11	ND	Pneumonia, n = 8‡
		A, C, G, J, *smeZ*, n = 7		Septic shock, n = 3‡
				Skin infection, n = 3
				UTI / PID, n = 3
				AOM, n = 1
***emm*4, n = 1**	E1	C, G, *ssa*, *smeZ*	ND	Cellulitis and septic shock
***emm*11, n = 3**	E6	C, G, H, n = 2	ND	Cellulitis, n = 2
		C, G, H, J, n = 1		Postoperative fever, n = 1
***emm*12, n = 6**	A-C4	C, G, H, n = 6	ND, n = 5	Fever, n = 2
			ERI, CLI, n = 1	Scarlet fever, n = 1
				Cellulitis, n = 1
				Postchemotherapy fever, n = 1
				Odynophagia, n = 1
***emm*33, n = 1**	D4	G, H, J, *smeZ*	TET	Septic arthritis
***emm*44, n = 1**	E3	A, G, J, *ssa*	ND	Pneumonia
***emm*49, n = 8**	E3	G, n = 5	TET, n = 7	Cellulitis, n = 6
		G, *ssa*, n = 1	ND, n = 1	Postchemotherapy fever, n = 1
		A, G, n = 1		Vascular ulcers, n = 1
		ND, n = 1		
***emm*58, n = 1**	E3	G, *ssa*	ERI, TET	Cellulitis
***emm*63, n = 1**	E6	*ssa*	TET	Cellulitis
***emm*75, n = 2**	E6	C, G, n = 2	ND	Cellulitis
***emm*77, n = 4**	E4	C, G, n = 2	iMLSB, TET, n = 4	Cellulitis, n = 3
		C, n = 2		Septic arthritis, n = 1
***emm*81, n = 1**	E6	G	TET	Cellulitis
***emm*82, n = 1**	E3	C, G, H	ND	Septic tenosynovitis
***emm*89, n = 8**	E4	C, G, n = 7	ND	Cellulitis, n = 3
		C, G, J, *smeZ*, n = 1		PID, n = 1
				Pneumonia, n = 3
				Prosthetic infection, n = 1
***emm*118, n = 1**	E3	G	TET	Cellulitis
***emm*132, n = 2**	Unknown	ND, n = 2	TET, n = 2	Cellulitis
***emm*145, n = 1**	Unknown	G, H	TET	PID
***emm*170, n = 1**	E5	C, G, H	ND	Septic arthritis

*AOM, acute otitis media; CLI, clindamycin; ERI, erythromycin; iMLSB, inducible macrolide-lincosamide-streptogramin B phenotype; ND, not detected; PID, pelvic inflammatory disease; TET, tetracyclin; UTI, urinary tract infection. †*emm* cluster classification as previously reported (*6*). ‡Exitus*: emm*1 (n = 3) and *emm*4 (n = 1).

Appendix Additional information about changes in group A *Streptococcus* *emm* types associated with invasive infections in adults, Spain, 2023.
