# Supplementary material for: Changes in Group A Streptococcus emm Types Associated with Invasive Infections in Adults, Spain, 2023
Source: Emerg Infect Dis. 2023 Nov;29(11):2390–2. doi: 10.3201/eid2911.230857 (PMC10617363; doi:10.3201/eid2911.230857)
Supplement: Appendix — Additional information about changes in group A Streptococcus emm types associated with invasive infections in adults, Spain, 2023. [file 23-0857-Techapp-s1.pdf]

Article DOI: https://doi.org/10.3201/eid2911.230857

EID cannot ensure accessibility for supplementary materials supplied by authors. Readers who have difficulty accessing supplementary content should contact the authors for assistance.

Changes in Group A *Streptococcus emm* Types Associated with Invasive Infections in Adults, Spain, 2023

Appendix

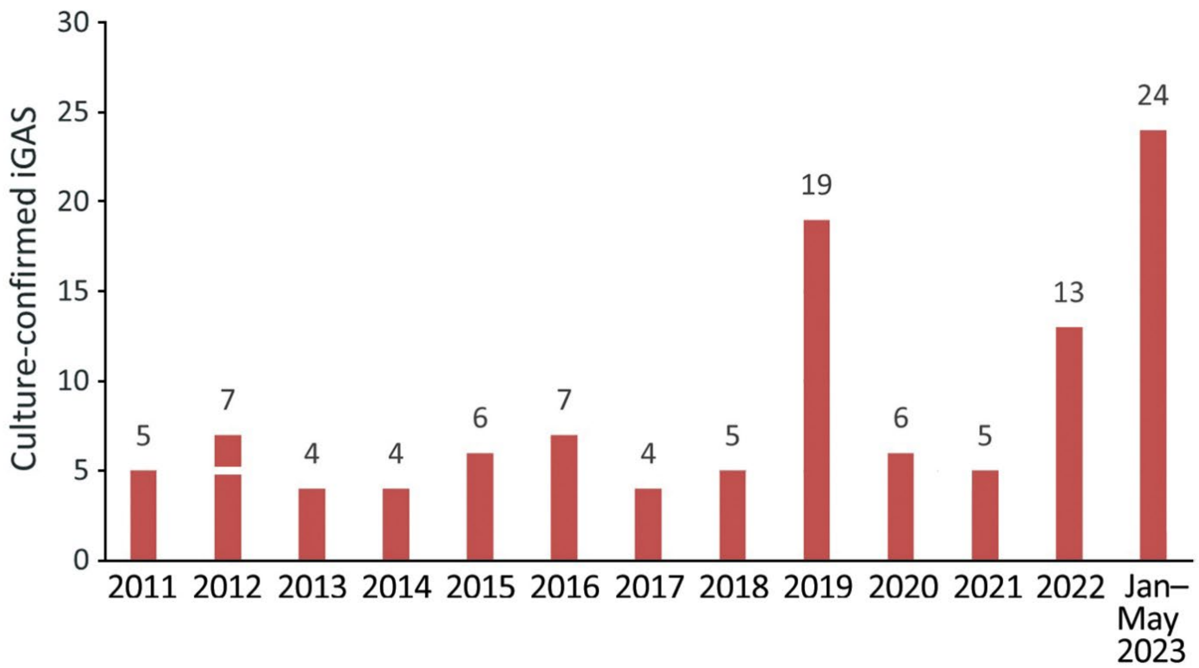

Appendix Figure. Culture-confirmed invasive group A *Streptococcus* infections per year, Lleida, Catalonia, Spain, January 2011–May 2023, n = 109.
